# Glucagon-Like Peptide-1 Receptor Agonists for Non-Alcoholic Fatty Liver Disease in Type 2 Diabetes: A Meta-Analysis

**DOI:** 10.3389/fendo.2021.609110

**Published:** 2021-04-09

**Authors:** Chloe Wong, Ming Hui Lee, Clyve Yu Leon Yaow, Yip Han Chin, Xin Lei Goh, Cheng Han Ng, Amanda Yuan Ling Lim, Mark Dhinesh Muthiah, Chin Meng Khoo

**Affiliations:** ^1^ Yong Loo Lin School of Medicine, National University of Singapore, Singapore, Singapore; ^2^ Department of Biological Sciences, Faculty of Science, National University of Singapore, Singapore, Singapore; ^3^ Department of Medicine, National University Hospital, Singapore, Singapore; ^4^ National University Centre for Organ Transplantation, National University Hospital, Singapore, Singapore

**Keywords:** glucagon-like peptide-1 receptor agonist, non-alcoholic fatty liver disease, type 2 diabetes mellitus, GLP-1RA, meta-analysis

## Abstract

**Objective:**

Non-alcoholic fatty liver disease is highly prevalent in patients with type 2 diabetes mellitus. Studies on glucagon-like peptide-1 receptor agonists for the treatment of non-alcoholic fatty liver disease have reported promising results. Despite this, there has been limited evidence of its efficacy in non-alcoholic fatty liver disease patients with type 2 diabetes mellitus. This meta-analysis examined existing evidence on the efficacy of glucagon-like peptide-1 receptor agonists on the management of non-alcoholic fatty liver disease in patients with type 2 diabetes mellitus.

**Methods:**

Medline, Embase and Cochrane Central Register of Controlled Trials (CENTRAL) were searched for articles discussing the efficacy of glucagon-like peptide-1 receptor agonists on non-alcoholic fatty liver disease in patients with type 2 diabetes mellitus. Values of standardized mean differences (SMD) and risk ratio (RR) were determined for continuous outcomes and dichotomous outcomes respectively.

**Results:**

8 studies involving 1,454 patients from 5 randomized controlled trials and 3 cohort studies were included in the analysis. Our analysis found significant improvements in hepatic fat content, liver biochemistry, body composition, glucose parameters, lipid parameters, insulin sensitivity and inflammatory markers following glucagon-like peptide-1 receptor agonist treatment. Glucagon-like peptide-1 receptor agonists significantly decreased hepatic fat content compared to metformin and insulin-based therapies. Glucagon-like peptide-1 receptor agonists also improved fibrosis markers, but this did not reach statistical significance.

**Conclusion:**

With a high prevalence of obesity and non-alcoholic fatty liver disease among patients with type 2 diabetes mellitus, glucagon-like peptide-1 receptor agonist treatment shows promise in improving both diabetes and non-alcoholic fatty liver disease phenotype.

## Introduction

Non-alcoholic fatty liver disease (NAFLD) is a condition characterized by hepatic steatosis and may be categorized into non-alcoholic fatty liver (NAFL) or non-alcoholic steatohepatitis (NASH). The global prevalence rate of NAFLD has been estimated to be 25% (1). The pathogenesis of NAFLD is a complex interplay of genetic, dietary and hormonal factors, with hyperinsulinemia and insulin resistance playing critical roles in NAFLD development (2, 3). NAFLD is a commonly found among patients with type 2 diabetes mellitus (T2DM), affecting as high as 60% of them (4).

T2DM and NAFLD share a bilateral pathogenic relationship. T2DM is a risk factor for the progression of NAFLD and its associated complications (5, 6), such as cirrhosis and hepatocellular carcinoma (7, 8). NAFLD increases the risk of T2DM, with higher risk among those NAFLD patients with advanced fibrosis (5). This mutual relationship would need a treatment that addresses both NAFLD and T2DM. Currently, there is no FDA-approved pharmacological therapy for NAFLD among patients with T2DM. While thiazolidinediones (TZD) have been used to treat patients with NASH (9, 10), there are concerns on its side effects such as weight gain, bone fractures and heart failure (11–13). The use of other pharmaceutical agents such as Sodium-glucose Co-transporter-2 inhibitors (SGLT2i) have also demonstrated a reduction in the hepatic fat content in some studies, however, more clinical trials are still needed to determine the efficacy of SGLT2i on NAFLD, including NASH (14).

Glucagon-like peptide-1 receptor agonists (GLP-1RA) are analogues of GLP-1 and fall under the class of incretin mimetics (15). GLP-1RA potentiate glucose-stimulated insulin secretion and inhibition of glucagon secretion, in addition to their effects on gastric satiety (15). GLP-1RA are one of the few glucose-lowering agents that results in weight loss (15), which have strong associations with improvements of hepatic steatosis (16). Apart from its effects on weight loss, GLP-1RA might play a direct role in improving hepatic steatosis through upregulations of insulin signaling pathways and fatty acid metabolism (17). Studies have shown a decrease in endogenous GLP-1 secretion in patients with NAFLD (18). Thus, the use of GLP-1RA in patients with NAFLD may be beneficial, and this is supported by the recent phase 2 LEAN study which reported histological improvements in steatohepatitis for patients with NASH (19). Recently, the GLP-1RA cardiovascular outcomes trials (CVOTs) have also reported cardiovascular protection among patients with diabetes mellitus, and this offers an attractive treatment option for patients with NAFLD (20). However, existing guidelines have cautioned against the use of GLP-1RA in patients with NAFLD due to limited evidence on its efficacy (16, 21, 22). This meta-analysis thus aims to examine the existing evidence on the efficacy of GLP1-RA in the management of NAFLD in patients with T2DM.

## Material and Methods

### Search Strategy

This meta-analysis was performed following the guidelines set by the Preferred Reporting Items for Systematic Reviews and Meta-analyses (23). Medline, Embase and Cochrane Central Register of Controlled Trials (CENTRAL) were searched for articles which reported the efficacy of GLP-1RA on NAFLD among patients with T2DM from inception to 21 June 2020. The terms “non-alcoholic fatty liver disease”, “type 2 diabetes mellitus” and “glucagon-like peptide-1” were included in the search strategy. The search strategy is detailed in the **Supplementary Material 1**.

### Eligibility and Selection Criteria

Articles were included if they examined the efficacy of GLP-1RA in the management of patients with T2DM and NAFLD in comparison with other treatment methods. Only original articles were included, while commentaries, editorials and reviews were excluded. The title-abstract sieve and full-text reviews were conducted by two independent authors and discrepancies were resolved through consensus or following discussion with a third independent author.

### Data Extraction

Relevant data was extracted by two independent authors onto a predefined template, which contained information relating to the study characteristics (sample size, country of origin and study design), therapeutic regimen of the treatment methods, as well as treatment outcomes (changes in body composition, metabolic parameters, adipokines and inflammatory markers, steatosis markers, fibrosis markers and liver biomarkers). Estimated values of the mean and standard deviation for continuous variables were derived through formulas when they were not provided (24, 25). Blinded checking of the data was performed by an independent third author and disparities were resolved through consensus.

### Statistical Analysis and Quality Assessment

Values of standardized mean differences (SMD) were determined for continuous outcomes to account for the different units of analysis (26). Risk ratio (RR) was determined for dichotomous outcomes. The analyses of continuous outcomes were conducted using STATA 16.1, with *p*<0.05 considered as statistically significant. The Newcastle Ottawa Scale (NOS) and Cochrane’s Risk of Bias (RoB2) tool were used in the quality assessment of cohort studies and randomized controlled trials respectively. The NOS assesses quality across the three domains of selection, comparability and outcome (27), while the RoB2 tool assesses quality across five domains including the randomization process, deviations from intended interventions, missing outcome data, outcome measurements and reporting (28).

## Results

### Baseline Characteristics

**Figure 1:**
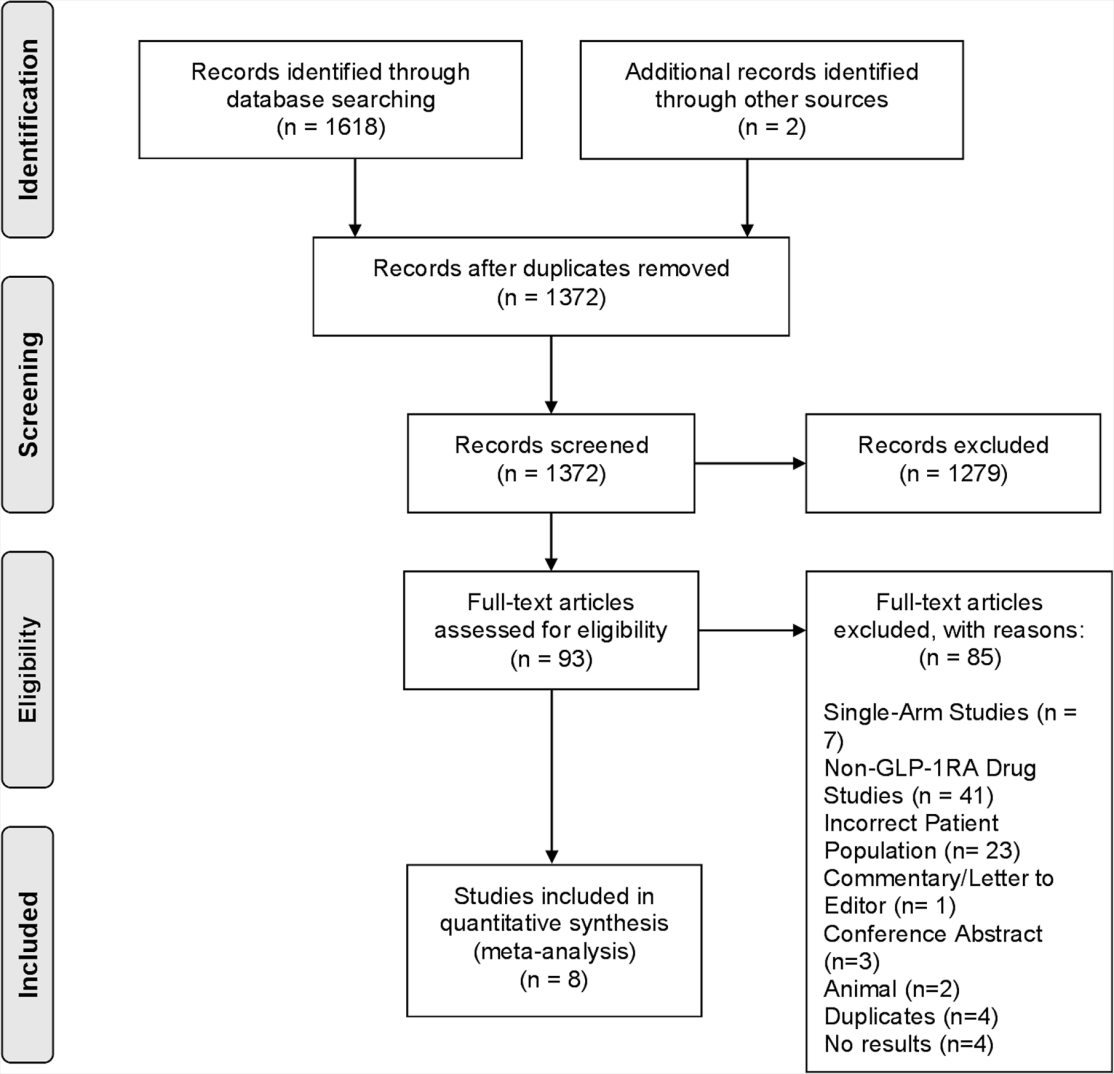
PRISMA flow diagram.

A total of 1,372 articles were obtained from the search strategy following duplicate removal. 93 articles subsequently underwent full-text review, of which 8 studies (29–36), including 5 randomized controlled trials (RCTs), fulfilled our eligibility criteria to be included in the final analysis (**Figure 1**).

**Figure 2:**
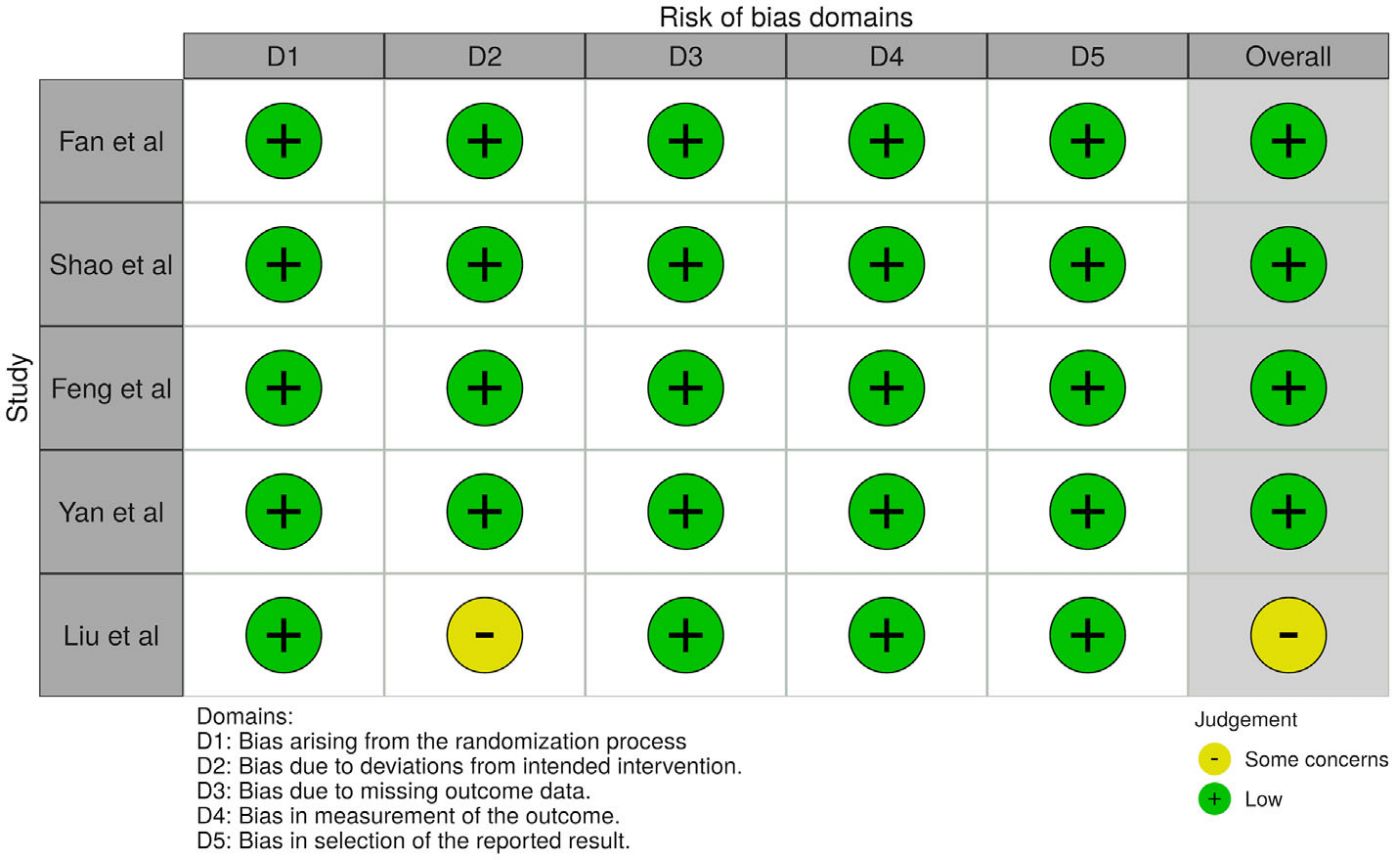
Risk of bias assessment.

There was a total of 1,454 patients included in our analysis, with all patients being clinically diagnosed with NAFLD and T2DM. This included 669 patients on GLP-1RA, 172 patients on metformin, 119 patients on insulin-based therapies (includes insulin secretagogues and insulin), 63 patients on DPP-4 inhibitors, 20 patients on TZD and 411 patients on conventional diabetic drug therapies (mainly metformin). GLP-1RA treatment comprised of liraglutide and exenatide, and the duration of treatment ranged between 12 to 52 weeks. All studied comparators will be classified under controls in the results. The glycated hemoglobin (HbA1c) ranged between 7.68% to 11.04%. The characteristics of patients are presented in **Table 1** and the quality assessment of included articles are found in **Supplementary Material 8** for cohort studies and **Figure 2** for RCTs.

**Table 1:** Summary of included articles.

Author, Year	Country	Study Design	GLP-1RA	Comparison	GLP-1RA (n)	Control (n)	Male (%)		Age		BMI (kg/m^2)		HbA1c (%)		Diagnosis Method
			Drug				GLP-1RA	Control	GLP-1RA	Control	GLP-1RA	Control	GLP-1RA	Control	
Ohki et al. (29)	Japan	Retrospective Study	Liraglutide	GLP-1RA vs. DPP-4 Inhibitors	26	36	69.23	80.56	56.07 ± 8.96	53.67 ± 16.00	29.67 ± 3.93	28.60 ± 4.59	8.40 ± 1.48	8.47 ± 1.48	Ultrasonography
Ohki et al. (29)	Japan	Retrospective Study	Liraglutide	GLP-1RA vs. TZD	26	20	69.23	70.00	56.07 ± 8.96	52.87 ± 9.85	29.67 ± 3.93	28.30 ± 5.11	8.40 ± 1.48	7.70 ± 1.19	Ultrasonography
Fan et al. (30)	China	RCT	Exenatide	GLP-1RA vs. Metformin	49	68	57.14	55.88	51.02 ± 10.10	54.68 ± 12.14	28.18 ± 1.86	27.61 ± 1.77	8.14 ± 0.51	8.09 ± 0.59	Ultrasonography
Shao et al. (31)	China	RCT	Exenatide	GLP-1RA vs. Insulin-based therapy	30	30	50.00	46.67	43.00 ± 4.10	42.00 ± 3.20	30.59 ± 1.09	30.29 ± 0.95	7.68 ± 0.57	7.59 ± 0.57	Guidelines for Diagnosis and Treatment of NAFLD, published in the Chinese Journal of Hepatology
Feng et al. (32)	China	RCT	Liraglutide	GLP-1RA vs. Insulin-based therapy	29	29	72.41	68.97	46.79 ± 9.69	48.07 ± 12.60	28.12 ± 3.02	27.85 ± 3.02	8.91 ± 1.72	9.03 ± 1.24	Ultrasonography
Feng et al. (32)	China	RCT	Liraglutide	GLP-1RA vs. Metformin	29	29	72.41	65.52	46.79 ± 9.69	46.31 ± 12.33	28.12 ± 3.02	26.82 ± 3.66	8.91 ± 1.72	9.36 ± 1.78	Ultrasonography
Tian et al. (33)	China	Prospective Study	Liraglutide	GLP-1RA vs. Metformin	52	75	59.62	57.33	58.50 ± 7.60	56.40 ± 8.40	28.18 ± 1.86	27.61 ± 1.77	8.14 ± 0.51	8.09 ± 0.59	B-mode Ultrasonic Scanning and according to Guidelines by the Fatty Liver and Alcoholic Liver Disease Group of the Chinese Medical Association in 2010
Zhang et al. (34)	China	Retrospective Study	Liraglutide	GLP-1RA vs. Conventional Drug Therapy	424	411	62.50	63.70	51.27 ± 8.16	52.05 ± 7.84	–	–	11.04 ± 1.08	11.53 ± 0.82	–
Yan et al. (35)	China	RCT	Liraglutide	GLP-1RA vs. DPP-4 Inhibitors	24	27	70.83	77.78	43.10 ± 9.70	45.70 ± 9.20	30.10 ± 3.30	29.70 ± 2.80	7.80 ± 1.40	7.60 ± 0.90	Practice guidelines by the American Association for the Study of Liver Diseases, American College of Gastroenterology, and the American Gastroenterological Association MRI-PDFF
Yan et al. (35)	China	RCT	Liraglutide	GLP-1RA vs. Insulin-based therapy	24	24	70.83	58.33	43.10 ± 9.70	45.6 ± 7.6	30.10 ± 3.30	29.60 ± 3.50	7.80 ± 1.40	7.70 ± 0.90	Practice guidelines by the American Association for the Study of Liver Diseases, American College of Gastroenterology, and the American Gastroenterological Association MRI-PDFF
Liu et al. (36)	China	RCT	Exenatide	GLP-1RA vs. Insulin-based therapy	35	36	54.29	52.78	47.63 ± 10.14	50.56 ± 11.78	28.49 ± 3.02	27.84 ± 3.10	8.32 ± 0.94	8.58 ± 0.91	Proton MRS ([^1^H] MRS]

BMI, Body Mass Index; HbA1c, Glycated hemoglobin; GLP-1RA, Glucagon-Like Peptide-1 Receptor Agonist; NA, Not Applicable; RCT, Randomized Controlled Trials.

### Changes in Hepatic Steatosis Markers

**Figure 3:**
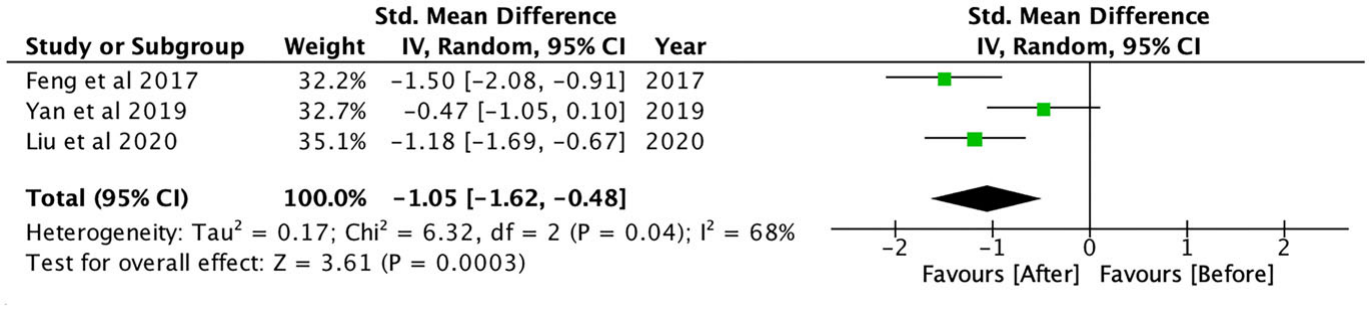
Hepatic fat content after GLP-1RA treatment.

**Figure 4:**
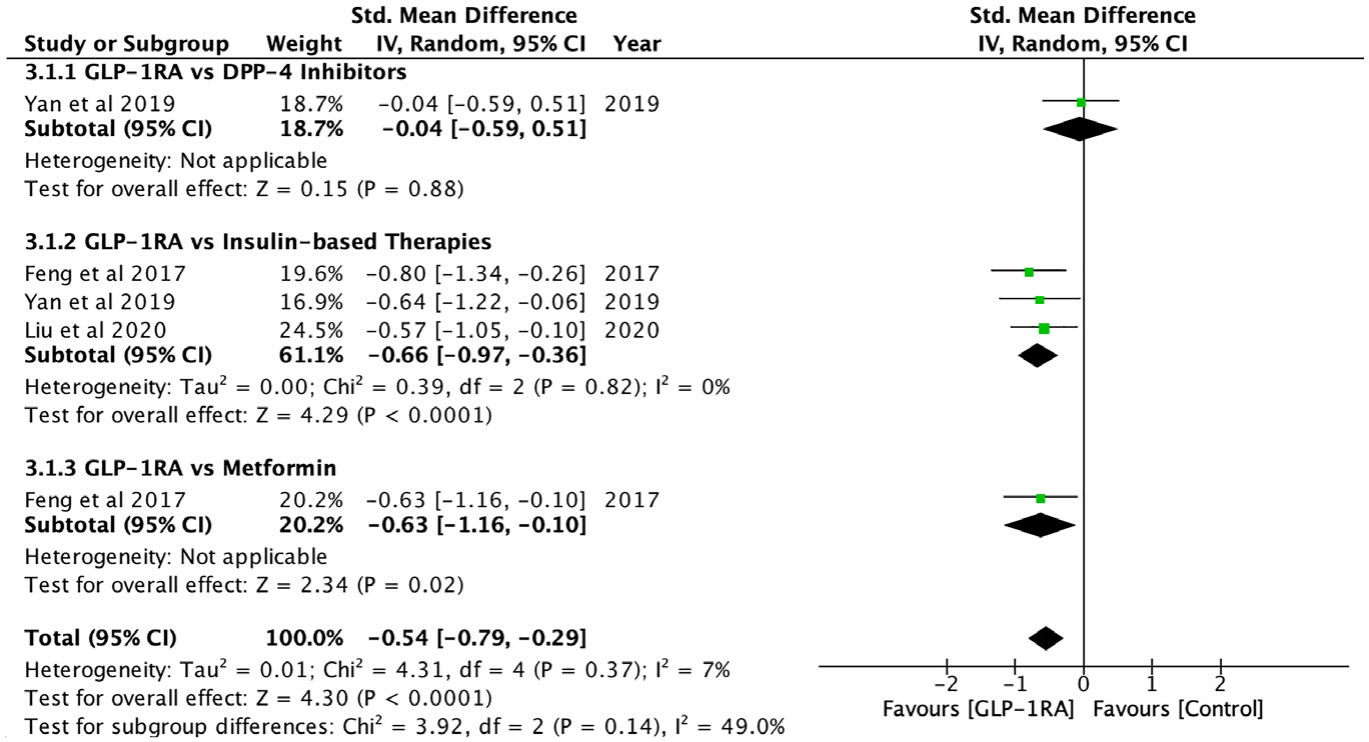
Hepatic fat content comparisons between GLP-1RA and controls.

Hepatic fat content, which was measured using magnetic resonance spectroscopy (MRS), magnetic resonance imaging proton density fat fraction (MRI-PDFF) and ultrasonography, was significantly reduced after GLP-1RA (SMD: -1.05; CI: -1.62 to -0.48; p<0.001, **Figure 3**), and when compared to controls (SMD: -0.54; CI: -0.79 to -0.29; p<0.001, **Figure 4**), metformin (SMD: -0.63, CI: -1.16 to -0.10; p=0.02) and insulin-based therapies (SMD: -0.66, CI: -0.97 to -0.36; p<0.001) (see **Supplementary Material 2**).

### Changes in Hepatic Fibrosis Markers

Information on the effect of GLP-1RA on hepatic fibrosis markers is less complete. Overall, GLP-1RA significantly reduced AST-to-Platelet Ratio Index (APRI) (SMD: -0.68; CI: -1.24 to -0.18; p=0.02), and when compared to DPP-4 inhibitors (SMD: -0.62; CI: -1.14 to -0.10; p=0.02). The AST/ALT ratio was significantly higher after GLP-1RA treatment (SMD: 1.65; CI: 1.33 to 1.97; p<0.001) and when compared to controls (SMD: 1.40; CI: 1.11 to 1.68; p<0.001) or metformin (SMD: 1.40; CI: 1.11 to 1.68; p<0.001) (see **Supplementary Material 3**).

### Changes in Liver Function Test

GLP-1RA significantly reduced total bilirubin (SMD: -5.83; CI: -7.01 to -4.66; p<0.001), aspartate aminotransferase (AST) (SMD: -1.46; CI: -2.22 to -0.79; p<0.001), alanine aminotransferase (ALT) (SMD: -1.69; CI: -2.32 to -1.07; p<0.001) and gamma-glutamyl transferase (GGT) (SMD: -2.10; CI: -3.16 to -1.04; p<0.001) (See **Supplementary Material 4**).

Compared to metformin, GLP-1RA resulted in significant decreases in ALT (SMD: -0.66; CI: -1.24 to -0.08; p=0.03) and GGT (SMD: -1.04; CI: -1.44 to -0.65; p<0.001). GLP-1RA also resulted in significantly lower ALT (SMD: -0.96; CI: -1.79 to -0.14; p=0.02) when compared to insulin-based therapies. On the contrary, TZD was more superior in the improvement in AST, ALT and GGT compared to GLP-1RA.

### Changes in Body Composition

**Figure 5:**
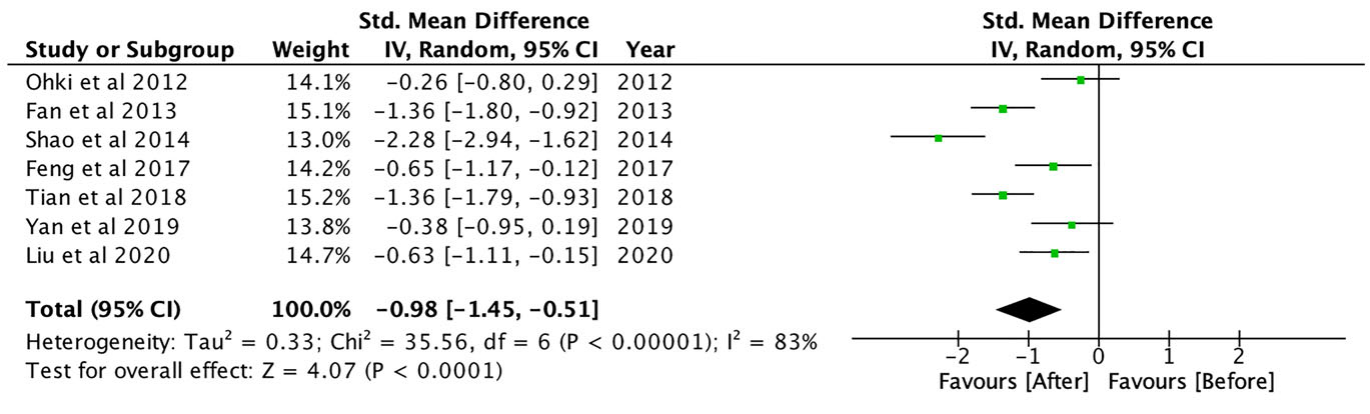
BMI after GLP-1RA treatment.

GLP-1RA significantly reduced body mass index (BMI) (SMD: -0.98; CI: -1.45 to -0.51; p<0.001, **Figure 5**), waist circumference (SMD: -1.31; CI: -2.47 to -0.15; p=0.03) and hip circumference (SMD: -2.39; CI: -3.06 to -1.73; p<0.001), waist-to-hip ratio (SMD: -0.69; CI: -1.23 to -0.16; p=0.01) and visceral adipose tissue (SMD: -0.57; CI: -0.94 to -0.20; p<0.01) (see **Supplementary Material 5**). Compared to controls, GLP-1RA significantly reduced BMI (SMD: -1.01, CI: -1.49 to -0.52; p<0.001), waist circumference (SMD: -1.22; CI: -2.22 to -0.22; p=0.02), hip circumference (SMD: -3.71; CI: -4.55 to -2.87; p<0.001), subcutaneous adipose tissue (SMD: -0.76; CI: -1.21 to -0.32; p<0.01) and visceral adipose tissue (SMD: -0.66; CI: -1.03 to -0.28; p<0.01). GLP-1RA was also more effective in reducing BMI compared to metformin (SMD: -0.58; CI: -0.81 to -0.34; p<0.001), TZD (SMD: -0.92; CI: -1.54 to -0.31; p<0.01), DPP-4 inhibitors (SMD: -0.44; CI: -0.82 to -0.07; p=0.02), or insulin-based therapies (SMD: -1.93; CI: -3.37 to -0.48; p<0.01). Compared to insulin-based therapies, GLP-1RA significantly reduced waist circumference (SMD: -1.84; CI: -3.43 to -0.25; p=0.02), subcutaneous adipose tissue (SMD: -0.90; CI: -1.58 to -0.22; p<0.01) and visceral adipose tissue (SMD: -0.80; CI: -1.19 to -0.41; p<0.001).

### Changes in Metabolic Parameters

GLP-1RA treatment resulted in a significant reduction in fasting glucose (SMD: -2.03; CI: -3.40 to -0.65; p<0.01), 2-hour postprandial glucose (SMD: -2.15; CI: -3.31 to -0.98; p<0.001) and HbA1c levels (SMD: -2.17; CI: -3.39 to -0.94; p<0.01). Compared to controls, GLP-1RA significantly reduced fasting glucose (SMD: -0.20; CI: -0.39 to -0.01; p=0.04), and 2-hour postprandial glucose (SMD: -0.56; CI: -0.75 to -0.38; p<0.001). Fasting glucose was significantly lower with GLP-1RA when compared to other DPP-4 inhibitors (SMD: -0.51; CI: -1.00 to -0.02; p=0.04). Compared to metformin, GLP-1RA had lower 2h-postprandial glucose (SMD: -0.62; CI: -0.85 to -0.38; p<0.001). GLP-1RA treatment also significantly decreased HOMA-IR (SMD: -1.04; CI: -1.35 to -0.73; p<0.001).

**Figure 6:**
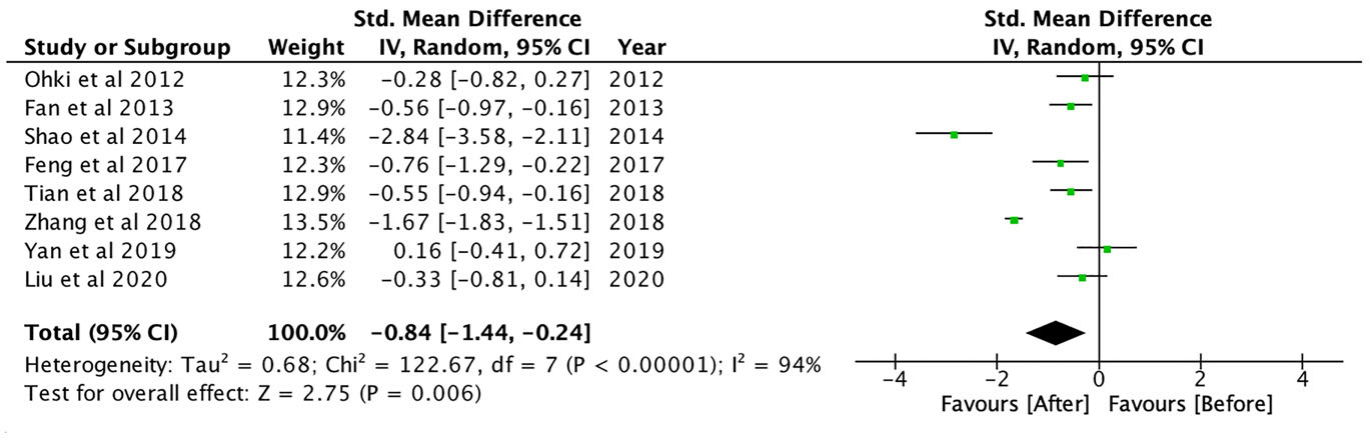
Triglycerides after GLP-1RA treatment.

GLP-1RA treatment also significantly reduced total cholesterol (SMD: -0.70; CI: -1.38 to -0.02; p=0.04), triglycerides (SMD: -0.84; CI: -1.44 to -0.24; p<0.01, **Figure 6**) and free fatty acid (FFA) levels (SMD: -0.62; CI: -1.10 to -0.14; p=0.01) (see **Supplementary Material 6**). GLP-1RA also resulted in significant reduction in systolic blood pressure (SMD: -0.66; CI: -0.96 to -0.35; p<0.001) and diastolic blood pressure (SMD: -0.33; CI: -0.59 to -0.07; p=0.01). Compared to controls, GLP-1RA had significantly lower systolic blood pressure (SMD: -0.25; CI: -0.46 to -0.04; p=0.02).

### Changes in Adipokines and Inflammatory Markers

GLP-1RA treatment significantly increased adiponectin levels (SMD: 0.84; CI: 0.58 to 1.09; p<0.001), and when compared to controls (SMD: 0.75; CI: 0.49 to 1.01; p<0.001), DPP-4 inhibitors (SMD: 0.61; CI: 0.04 to 1.17; p=0.04), insulin-based therapies (SMD: 1.32; CI: 0.69 to 1.95; p<0.001) or metformin (SMD: 0.66; CI: 0.40 to 0.93; p<0.001). GLP-1RA significantly decreased inflammatory biomarkers, cytokeratin-18 (CK-18) (SMD: -1.28; CI: -1.70 to -0.86; p<0.001), interleukin-6 (IL-6) (SMD: -0.74; CI: -1.33 to -0.16; p=0.01) and C-reactive protein (CRP) levels (SMD: -2.02; CI: -2.36 to -1.68; p<0.001) (see **Supplementary Material 7**). Compared to controls or metformin, GLP-1RA significantly reduced CRP levels (Controls, SMD: -0.50; CI: -0.76 to -0.24; p<0.001, Metformin, SMD: -0.50; CI: -0.76 to -0.24; p<0.001). Compared to insulin-based therapies, GLP-1RA significantly decreased IL-6 levels (SMD: -0.75; CI: -1.34 to -0.17; p=0.01).

## Discussion

This meta-analysis demonstrated that GLP1-RA treatment improved hepatic steatosis, liver function, lipid, glucose, inflammatory and insulin sensitivity markers, which indicates a synergistic treatment approach for patients with T2DM and NAFLD. NAFLD is frequently asymptomatic until late stages of the disease (37), which may lead to a delay in the diagnosis of NAFLD. Upon diagnosis of NAFLD, there are no clear therapeutic guidelines or management recommendations for these patients besides weight loss and dietary recommendations (16, 21). A previous meta-analysis found that GLP1-RA treatment could potentially improve liver histology in NAFLD patients (38). Hence, the results of this meta-analysis add to this knowledge by demonstrating the efficacy of GLP-1RA on improving NAFLD and liver fibrosis in patients with T2DM.

In our analysis, GLP-1RA treatment had favorable outcomes in the improvement of hepatic steatosis, as measured by magnetic resonance spectroscopy, magnetic resonance imaging proton density fat fraction and ultrasonography. GLP-1RA also demonstrated better efficacy in lowering hepatic fat content than metformin or insulin-based therapies. It has been shown that GLP-1RA improve β-cell function, enhances hepatic insulin sensitivity and modulates genes involved in fatty acid oxidation and *de novo* lipogenesis in the liver (17, 39–42). GLP-1RA may also reduce hepatic fat content through its effect on weight loss (39, 43). Sulfonylureas and insulin treatment are known to be associated with weight gain (44), while GLP-1RA induce weight loss (45), which could explain the advantage of GLP-1RA over insulin-based therapies as treatment for NAFLD.

Weight loss is the cornerstone therapy for NAFLD, and weight management is strongly recommended for all patients with NAFLD (46). The association between weight loss and reduction in hepatic fat and improvement in liver histology is one of the key reasons for the recommendation of weight loss across various guidelines in the management of NAFLD (16, 21, 38, 47, 48). Weight loss has also been significantly associated with enhanced insulin sensitivity (49), which has been demonstrated in our findings with a observed reduction of HOMA-IR and elevation of adiponectin with the use of GLP-1RA. As strong associations have been established between NAFLD, T2DM and insulin resistance (5, 41), the increase in insulin sensitivity with the use of GLP-1RA can have a valuable synergistic effect on improvement of both NAFLD and T2DM. However, the efficacy of weight loss *via* lifestyle and diet interventions may be limited in the case of poor patient adherence and weight rebound in the long-term (16, 21). In this meta-analysis, GLP-1RA treatment leads to significant improvements in body composition such as reduction in body weight, BMI, waist and hip circumference, which reinforces the advantage of using GLP-1RA as part of the treatment strategy for patients with NAFLD. Of the eight included studies, three assessed changes in hepatic fat content following GLP1-RA treatment, of which all reported correlations between weight loss and decreases in hepatic fat content (32, 35, 36), lending support for the role of weight loss on hepatic steatosis. It has yet to be determined whether GLP-1RA-induced weight loss or the direct effects of GLP-1RA on the hepatocytes plays the major role in the reduction of hepatic steatosis.

The FIB-4 index, NAFLD Fibrosis Score (NFS) and APRI could be used to indicate severity of hepatic fibrosis. These biomarkers have been shown to predict the prognosis of liver fibrosis with varying degrees of sensitivity and specificity (50). This study showed that there was improvement in APRI and FIB-4 index after GLP-1RA treatment, although the changes in FIB-4 index did not reach statistical significance. The mechanism of GLP-1RA on hepatic fibrosis can be attributed partly to the decrease in pro-inflammatory adipokines such as leptin, (monocyte chemoattractant protein-1and resistin, which are associated with the progression to NASH and fibrosis (51). In addition, the LEAN trial also suggested that liraglutide should be started early to prevent progression of NAFLD, and might not be useful in NASH patients with advanced fibrosis (19). Consistent with the improvement in the liver fat content, steatosis and fibrosis biomarkers, we also found that GLP-1RA treatment improved liver function markers such as AST, ALT and GGT. Apart from fibrosis markers and liver function tests, the observed reductions in inflammatory markers such as CRP and CK-18 could be attributed to downregulation of proinflammatory responses and hepatic inflammation (52). Additionally, considering CK-18 as a marker of severity in NASH, GLP-1RA could possibly benefit patients with NASH (53, 54).

Despite the benefits of GLP-1RA on the treatment of NAFLD, we are also cognizant of the adverse effects of GLP-1RA treatment, and in particular, the gastrointestinal side effects such as nausea, vomiting and diarrhea and hypoglycemia. GLP-1RA should be avoided in patients at risk for pancreatitis or with previous history of pancreatitis (55). GLP1-RA are also contraindicated in patients with a personal or family history of medullary thyroid cancer and in patients with Multiple Endocrine Neoplasia syndrome type 2 (MEN 2) as they have been shown to cause thyroid C-cell tumors in rodents (56, 57).

### Limitations

There are several limitations in this analysis. The included studies utilized magnetic resonance spectroscopy, magnetic resonance imaging proton density fat fraction and ultrasonography in the assessment and quantification of hepatic steatosis, rather than liver biopsy which is a more accurate reflection of liver histology as defined by regulatory agencies from the United States and Europe and is currently required for phase 2B and phase 3 clinical trials for NAFLD (58, 59). In addition, the criteria for the ultrasonography diagnosis of fatty liver were different for studies included in this meta-analysis. Fan et al. mentioned that the fatty liver was diagnosed based on the criteria of: 1) enlarged liver, together with the increase and intensification in echoes and bright spots, 2) unclear or absent intrahepatic blood vessels and 3) reduced or attenuated deep echoes. Feng et al. however, mentioned that the intrahepatic fat was quantified using a quantitative assessment method and standardized US hepatic/renal ratio and hepatic attenuation rate. While the methodology of ultrasonography may vary, liver ultrasonography generally correlated well with steatosis at liver biopsy, but less so for fibrosis and NASH (60). In addition, the lack of sufficient trials reporting biopsy-proven efficacy of GLP-1RA could be a potential contributing factor for why GLP-1RA have not been recommended as a therapy of choice by the regulatory agencies (16). Despite this, magnetic resonance imaging proton density fat fraction and magnetic resonance spectroscopy technologies have been proven to be accurate substitutions for liver biopsy (61), and can accurately quantify hepatic steatosis. Furthermore, due to the lack of placebo-controlled studies which fit the inclusion criteria, the study did not include placebo-controlled studies. Another limitation in our analysis is that we were unable to account for the duration of NAFLD in patients, which may have influenced treatment response, as reported by the LEAN trial (19). The lack of statistical significance observed in the fibrosis markers could be partially attributed to the insufficient follow-up time, as the long disease progression for liver fibrosis may require longer treatment durations for observations of clinically significant fibrosis reversal (62). Lastly, only four studies reported on adverse events from GLP1-RA treatment. The long-term safety profile of GLP-1RA need to be verified in further studies.

## Conclusion

Among patients with T2DM and NAFLD, GLP-1RA treatment improves body composition, glycemic control, insulin sensitivity, and biomarkers of inflammation and hepatic steatosis. The beneficial effects of GLP-1RA on NAFLD is likely to be contributed indirectly by weight loss and directly by its effect on liver hepatocytes and hepatic lipid metabolism. Longer term studies are needed to determine the effects of GLP-1RA on liver fibrosis in patients with NAFLD and T2DM.

## Data Availability Statement

The original contributions presented in the study are included in the article/**Supplementary Material**. Further inquiries can be directed to the corresponding author.

## Author Contributions

CW – literature search, data extraction, data analysis and interpretation, drafting and critical revisions of the manuscript. ML - literature search, data extraction, data analysis and interpretation, drafting and critical revisions of the manuscript. CY – literature search, data extraction, and data analysis and interpretation. YC – data analysis and interpretation, critical revision of article and final approval. XG – drafting of article and critical revision of article. CN – study conception, literature search, methodology, data analysis and interpretation, critical revision of article and final approval. AL – critical revision of the article and final approval. MM – data interpretation, critical revision of the article and final approval. CK – study conception, methodology, data interpretation, critical revision of article and final approval. All authors contributed to the article and approved the submitted version.

## Conflict of Interest

The authors declare that the research was conducted in the absence of any commercial or financial relationships that could be construed as a potential conflict of interest.

## References

[1] Younossi ZM Koenig AB Abdelatif D Fazel Y Henry L Wymer M. Global epidemiology of nonalcoholic fatty liver disease-Meta-analytic assessment of prevalence, incidence, and outcomes. Hepatology (2016) 64(1):73–84. 10.1002/hep.28431 26707365

[2] Carr RM Oranu A Khungar V. Nonalcoholic Fatty Liver Disease: Pathophysiology and Management. Gastroenterol Clin North Am (2016) 45(4):639–52. 10.1016/j.gtc.2016.07.003 PMC5127277 27837778

[3] Cobbina E Akhlaghi F. Non-alcoholic fatty liver disease (NAFLD) - pathogenesis, classification, and effect on drug metabolizing enzymes and transporters. Drug Metab Rev (2017) 49(2):197–211. 10.1080/03602532.2017.1293683 28303724 PMC5576152

[4] Dai W Ye L Liu A Wen SW Deng J Wu X. Prevalence of nonalcoholic fatty liver disease in patients with type 2 diabetes mellitus: A meta-analysis. Med (Baltimore) (2017) 96(39):e8179. 10.1097/md.0000000000008179 PMC5626318 28953675

[5] Lonardo A Nascimbeni F Mantovani A Targher G. Hypertension, diabetes, atherosclerosis and NASH: Cause or consequence? J Hepatol (2018) 68(2):335–52. 10.1016/j.jhep.2017.09.021 29122390

[6] McPherson S Hardy T Henderson E Burt AD Day CP Anstee QM. Evidence of NAFLD progression from steatosis to fibrosing-steatohepatitis using paired biopsies: implications for prognosis and clinical management. J Hepatol (2015) 62(5):1148–55. 10.1016/j.jhep.2014.11.034 25477264

[7] Reeves HL Zaki MY Day CP. Hepatocellular Carcinoma in Obesity, Type 2 Diabetes, and NAFLD. Dig Dis Sci (2016) 61(5):1234–45. 10.1007/s10620-016-4085-6 26921078

[8] Sass DA Chang P Chopra KB. Nonalcoholic fatty liver disease: a clinical review. Dig Dis Sci (2005) 50(1):171–80. 10.1007/s10620-005-1267-z 15712657

[9] Cusi K Orsak B Bril F Lomonaco R Hecht J Ortiz-Lopez C. Long-Term Pioglitazone Treatment for Patients With Nonalcoholic Steatohepatitis and Prediabetes or Type 2 Diabetes Mellitus: A Randomized Trial. Ann Intern Med (2016) 165(5):305–15. 10.7326/m15-1774 27322798

[10] Sanyal AJ Chalasani N Kowdley KV McCullough A Diehl AM Bass NM. Pioglitazone, vitamin E, or placebo for nonalcoholic steatohepatitis. N Engl J Med (2010) 362(18):1675–85. 10.1056/NEJMoa0907929 PMC2928471 20427778

[11] Balas B Belfort R Harrison SA Darland C Finch J Schenker S. Pioglitazone treatment increases whole body fat but not total body water in patients with non-alcoholic steatohepatitis. J Hepatol (2007) 47(4):565–70. 10.1016/j.jhep.2007.04.013 17560678

[12] Hernandez AV Usmani A Rajamanickam A Moheet A. Thiazolidinediones and risk of heart failure in patients with or at high risk of type 2 diabetes mellitus: a meta-analysis and meta-regression analysis of placebo-controlled randomized clinical trials. Am J Cardiovasc Drugs (2011) 11(2):115–28. 10.2165/11587580-000000000-00000 21294599

[13] Schwartz AV Sellmeyer DE Vittinghoff E Palermo L Lecka-Czernik B Feingold KR. Thiazolidinedione use and bone loss in older diabetic adults. J Clin Endocrinol Metab (2006) 91(9):3349–54. 10.1210/jc.2005-2226 PMC1563497 16608888

[14] Wong C Yaow CYL Ng CH Chin YH Low YF Lim AYL. Sodium-Glucose Co-Transporter 2 Inhibitors for Non-Alcoholic Fatty Liver Disease in Asian Patients With Type 2 Diabetes: A Meta-Analysis. Front Endocrinol (2021) 11:609135. 10.3389/fendo.2020.609135 PMC7905212 33643221

[15] Dhir G Cusi K. Glucagon like peptide-1 receptor agonists for the management of obesity and non-alcoholic fatty liver disease: a novel therapeutic option. J Investig Med (2018) 66(1):7–10. 10.1136/jim-2017-000554 28918389

[16] Chalasani N Younossi Z Lavine JE Charlton M Cusi K Rinella M. The diagnosis and management of nonalcoholic fatty liver disease: Practice guidance from the American Association for the Study of Liver Diseases. Hepatology (2018) 67(1):328–57. 10.1002/hep.29367 28714183

[17] Gupta NA Mells J Dunham RM Grakoui A Handy J Saxena NK. Glucagon-like peptide-1 receptor is present on human hepatocytes and has a direct role in decreasing hepatic steatosis in vitro by modulating elements of the insulin signaling pathway. Hepatology (2010) 51(5):1584–92. 10.1002/hep.23569 PMC2862093 20225248

[18] Bernsmeier C Meyer-Gerspach AC Blaser LS Jeker L Steinert RE Heim MH. Glucose-induced glucagon-like Peptide 1 secretion is deficient in patients with non-alcoholic fatty liver disease. PloS One (2014) 9(1):e87488. 10.1371/journal.pone.0087488 24489924 PMC3906180

[19] Armstrong MJ Gaunt P Aithal GP Barton D Hull D Parker R. Liraglutide safety and efficacy in patients with non-alcoholic steatohepatitis (LEAN): a multicentre, double-blind, randomised, placebo-controlled phase 2 study. Lancet (2016) 387(10019):679–90. 10.1016/s0140-6736(15)00803-x 26608256

[20] Stepanova M Rafiq N Makhlouf H Agrawal R Kaur I Younoszai Z. Predictors of all-cause mortality and liver-related mortality in patients with non-alcoholic fatty liver disease (NAFLD). Dig Dis Sci (2013) 58(10):3017–23. 10.1007/s10620-013-2743-5 23775317

[21] European Association for the Study of the Liver (EASL), European Association for the Study of Diabetes (EASD), European Association for the Study of Obesity (EASO) EASL-EASD-EASO Clinical Practice Guidelines for the management of non-alcoholic fatty liver disease. J Hepatol (2016) 64(6):1388–402. 10.1016/j.jhep.2015.11.004 27062661

[22] Kalogirou M Sinakos E. Treating nonalcoholic steatohepatitis with antidiabetic drugs: Will GLP-1 agonists end the struggle? World J Hepatol (2018) 10(11):790–4. 10.4254/wjh.v10.i11.790 PMC6280165 30533179

[23] Moher D Liberati A Tetzlaff J Altman DG. Preferred reporting items for systematic reviews and meta-analyses: the PRISMA statement. PloS Med (2009) 6(7):e1000097. 10.1371/journal.pmed.1000097 19621072 PMC2707599

[24] Hozo SP Djulbegovic B Hozo I. Estimating the mean and variance from the median, range, and the size of a sample. BMC Med Res Methodol (2005) 5:13. 10.1186/1471-2288-5-13 15840177 PMC1097734

[25] Wan X Wang W Liu J Tong T. Estimating the sample mean and standard deviation from the sample size, median, range and/or interquartile range. BMC Med Res Methodol (2014) 14:135. 10.1186/1471-2288-14-135 25524443 PMC4383202

[26] Takeshima N Sozu T Tajika A Ogawa Y Hayasaka Y Furukawa TA. Which is more generalizable, powerful and interpretable in meta-analyses, mean difference or standardized mean difference? BMC Med Res Methodol (2014) 14:30. 10.1186/1471-2288-14-30 24559167 PMC3936842

[27] Wells G Shea B O’connell D Peterson J Welch V Losos M. The Newcastle-Ottawa Scale (NOS) for Assessing the Quality of Nonrandomised Studies in Meta-Analyses (2014). Available at: http://www.ohri.ca/programs/clinical_epidemiology/oxford.asp (Accessed August 23, 2020).

[28] Higgins JPT Altman DG Gøtzsche PC Jüni P Moher D Oxman AD. The Cochrane Collaboration’s tool for assessing risk of bias in randomised trials. BMJ (2011) 343:d5928. 10.1136/bmj.d5928 22008217 PMC3196245

[29] Ohki T Isogawa A Iwamoto M Ohsugi M Yoshida H Toda N. The effectiveness of liraglutide in nonalcoholic fatty liver disease patients with type 2 diabetes mellitus compared to sitagliptin and pioglitazone. ScientificWorldJournal (2012) 2012:496453. 10.1100/2012/496453 22927782 PMC3425807

[30] Fan H Pan Q Xu Y Yang X. Exenatide improves type 2 diabetes concomitant with non-alcoholic fatty liver disease. Arq Bras Endocrinol Metabol (2013) 57(9):702–8. 10.1590/s0004-27302013000900005 24402015

[31] Shao N Kuang HY Hao M Gao XY Lin WJ Zou W. Benefits of exenatide on obesity and non-alcoholic fatty liver disease with elevated liver enzymes in patients with type 2 diabetes. Diabetes Metab Res Rev (2014) 30(6):521–9. 10.1002/dmrr.2561 24823873

[32] Feng W Gao C Bi Y Wu M Li P Shen S. Randomized trial comparing the effects of gliclazide, liraglutide, and metformin on diabetes with non-alcoholic fatty liver disease. J Diabetes (2017) 9(8):800–9. 10.1111/1753-0407.12555 28332301

[33] Tian F Zheng Z Zhang D He S Shen J. Efficacy of liraglutide in treating type 2 diabetes mellitus complicated with non-alcoholic fatty liver disease. Biosci Rep (2018) 38(6):BSR2018130. 10.1042/bsr20181304 PMC6435530 30473540

[34] Zhang Z Qi Y Kong W Jin Q Wang X Dong Y. Efficacy and Clinical Value of Liraglutide for Treatment of Diabetes Mellitus Complicated by Non-Alcoholic Fatty Liver Disease. Med Sci Monit (2018) 24:7399–404. 10.12659/msm.911062 PMC6199821 30325900

[35] Yan J Yao B Kuang H Yang X Huang Q Hong T. Liraglutide, Sitagliptin, and Insulin Glargine Added to Metformin: The Effect on Body Weight and Intrahepatic Lipid in Patients With Type 2 Diabetes Mellitus and Nonalcoholic Fatty Liver Disease. Hepatology (2019) 69(6):2414–26. 10.1002/hep.30320 PMC6594101 30341767

[36] Liu L Yan H Xia M Zhao L Lv M Zhao N. Efficacy of exenatide and insulin glargine on nonalcoholic fatty liver disease in patients with type 2 diabetes. Diabetes Metab Res Rev (2020) 36(5):e3292. 10.1002/dmrr.3292 31955491

[37] Dyson JK Anstee QM McPherson S. Non-alcoholic fatty liver disease: a practical approach to diagnosis and staging. Frontline Gastroenterol (2014) 5(3):211–8. 10.1136/flgastro-2013-100403 PMC4078666 25018867

[38] Dong Y Lv Q Li S Wu Y Li L Li J. Efficacy and safety of glucagon-like peptide-1 receptor agonists in non-alcoholic fatty liver disease: A systematic review and meta-analysis. Clin Res Hepatol Gastroenterol (2017) 41(3):284–95. 10.1016/j.clinre.2016.11.009 28065744

[39] Liu J Wang G Jia Y Xu Y. GLP-1 receptor agonists: effects on the progression of non-alcoholic fatty liver disease. Diabetes Metab Res Rev (2015) 31(4):329–35. 10.1002/dmrr.2580 25066109

[40] Svegliati-Baroni G Saccomanno S Rychlicki C Agostinelli L De Minicis S Candelaresi C. Glucagon-like peptide-1 receptor activation stimulates hepatic lipid oxidation and restores hepatic signalling alteration induced by a high-fat diet in nonalcoholic steatohepatitis. Liver Int (2011) 31(9):1285–97. 10.1111/j.1478-3231.2011.02462.x 21745271

[41] Bhatt HB Smith RJ. Fatty liver disease in diabetes mellitus. Hepatobiliary Surg Nutr (2015) 4(2):101–8. 10.3978/j.issn.2304-3881.2015.01.03 PMC4405411 26005676

[42] Shyangdan DS Royle P Clar C Sharma P Waugh N Snaith A. Glucagon-like peptide analogues for type 2 diabetes mellitus. Cochrane Database Syst Rev (2011) 2011(10):Cd006423. 10.1002/14651858.CD006423.pub2 PMC6486297 21975753

[43] Bradley DP Kulstad R Schoeller DA. Exenatide and weight loss. Nutrition (2010) 26(3):243–9. 10.1016/j.nut.2009.07.008 20152707

[44] Hazlehurst JM Woods C Marjot T Cobbold JF Tomlinson JW. Non-alcoholic fatty liver disease and diabetes. Metabolism (2016) 65(8):1096–108. 10.1016/j.metabol.2016.01.001 PMC4943559 26856933

[45] Meloni AR DeYoung MB Lowe C Parkes DG. GLP-1 receptor activated insulin secretion from pancreatic β-cells: mechanism and glucose dependence. Diabetes Obes Metab (2013) 15(1):15–27. 10.1111/j.1463-1326.2012.01663.x 22776039 PMC3556522

[46] Cusi K. Time to Include Nonalcoholic Steatohepatitis in the Management of Patients With Type 2 Diabetes. Diabetes Care (2020) 43(2):275–9. 10.2337/dci19-0064 31959644

[47] Koutoukidis DA Astbury NM Tudor KE Morris E Henry JA Noreik M. Association of Weight Loss Interventions With Changes in Biomarkers of Nonalcoholic Fatty Liver Disease: A Systematic Review and Meta-analysis. JAMA Intern Med (2019) 179(9):1262–71. 10.1001/jamainternmed.2019.2248 PMC6604126 31260026

[48] Nascimbeni F Pais R Bellentani S Day CP Ratziu V Loria P. From NAFLD in clinical practice to answers from guidelines. J Hepatol (2013) 59(4):859–71. 10.1016/j.jhep.2013.05.044 23751754

[49] Clamp LD Hume DJ Lambert EV Kroff J. Enhanced insulin sensitivity in successful, long-term weight loss maintainers compared with matched controls with no weight loss history. Nutr Diabetes (2017) 7(6):e282. 10.1038/nutd.2017.31 28628125 PMC5519190

[50] Siddiqui MS Patidar KR Boyett S Luketic VA Puri P Sanyal AJ. Performance of non-invasive models of fibrosis in predicting mild to moderate fibrosis in patients with non-alcoholic fatty liver disease. Liver Int (2016) 36(4):572–9. 10.1111/liv.13054 26713759

[51] Gastaldelli A Marchesini G. Time for Glucagon like peptide-1 receptor agonists treatment for patients with NAFLD? J Hepatol (2016) 64(2):262–4. 10.1016/j.jhep.2015.11.031 26643784

[52] Montandon SA Somm E Vito CD Jornayvaz FR. 1887-P: The GLP-1 Receptor Agonist Liraglutide Improves Hepatic Inflammation and Fibrosis in a Mouse Model of NASH. Diabetes (2019) 68(Supplement 1):1887–P. 10.2337/db19-1887-P

[53] Feldstein AE Wieckowska A Lopez AR Liu YC Zein NN McCullough AJ. Cytokeratin-18 fragment levels as noninvasive biomarkers for nonalcoholic steatohepatitis: a multicenter validation study. Hepatology (2009) 50(4):1072–8. 10.1002/hep.23050 PMC2757511 19585618

[54] Lee YS Jun HS. Anti-Inflammatory Effects of GLP-1-Based Therapies beyond Glucose Control. Mediators Inflammation (2016) 2016:3094642. 10.1155/2016/3094642 PMC4823510 27110066

[55] Prasad-Reddy L Isaacs D. A clinical review of GLP-1 receptor agonists: efficacy and safety in diabetes and beyond. Drugs Context (2015) 4:212283. 10.7573/dic.212283 26213556 PMC4509428

[56] Food and Drug Administration. VICTOZA (liraglutide) injection (2017). Available at: https://www.accessdata.fda.gov/drugsatfda_docs/label/2017/022341s027lbl.pdf (Accessed 18 September 2020).

[57] Food and Drug Administration. OZEMPIC (semaglutide) Injection (2017). Available at: https://www.accessdata.fda.gov/drugsatfda_docs/label/2017/209637lbl.pdf (Accessed 18 September 2020).

[58] Food and Drug Administration. Noncirrhotic Nonalcoholic Steatohepatitis With Liver Fibrosis: Developing Drugs for Treatment. (2018). Available at: https://www.fda.gov/media/119044/download (Accessed 18 September 2020).

[59] Siddiqui MS Harrison SA Abdelmalek MF Anstee QM Bedossa P Castera L. Case definitions for inclusion and analysis of endpoints in clinical trials for nonalcoholic steatohepatitis through the lens of regulatory science. Hepatology (2018) 67(5):2001–12. 10.1002/hep.29607 PMC5906171 29059456

[60] Ballestri S Romagnoli D Nascimbeni F Francica G Lonardo A. Role of ultrasound in the diagnosis and treatment of nonalcoholic fatty liver disease and its complications. Expert Rev Gastroenterol Hepatol (2015) 9(5):603–27. 10.1586/17474124.2015.1007955 25694178

[61] Pirmoazen A Khurana A El Kaffas A Kamaya A. Quantitative ultrasound approaches for diagnosis and monitoring hepatic steatosis in nonalcoholic fatty liver disease. Theranostics (2020) 10:4277–89. 10.7150/thno.40249 PMC7086372 32226553

[62] Ellis EL Mann DA. Clinical evidence for the regression of liver fibrosis. J Hepatol (2012) 56(5):1171–80. 10.1016/j.jhep.2011.09.024 22245903

