# Supplementary material for: Glucagon-Like Peptide-1 Receptor Agonists for Non-Alcoholic Fatty Liver Disease in Type 2 Diabetes: A Meta-Analysis
Source: Front Endocrinol (Lausanne). 2021 Apr 9;12:609110. doi: 10.3389/fendo.2021.609110 (PMC8063104; doi:10.3389/fendo.2021.609110)
Supplement: Supplementary file 1 [file DataSheet_1.docx]

**Glucagon-like Peptide-1 Receptor Agonists for Non-alcoholic Fatty Liver Disease in Type 2 Diabetes: A Meta-analysis**

Chloe Wong^ψ1^, Ming Hui Lee^ψ2^, Clyve Yu Leon Yaow^1^, Yip Han Chin^1^, Xin Lei Goh^1^, Cheng Han Ng^1^, Amanda Yuan Ling Lim^1,3^, Mark Dhinesh Muthiah^1,3,4^, Chin Meng Khoo*^1,3^

^1^Yong Loo Lin School of Medicine, National University of Singapore, Singapore, Singapore, 10 Medical Dr, Singapore 117597

^2^Department of Biological Sciences, Faculty of Science, National University of Singapore, Singapore

^3^Department of Medicine, National University Hospital, Singapore

^4^National University Centre for Organ Transplantation, National University Hospital, Singapore

^ψ^The authors had equal contribution

***Correspondence:**

*Khoo Chin Meng*

Yong Loo Lin School of Medicine,

10 Medical Dr, Singapore 117597

Email: [mdckcm@nus.edu.sg](mailto:mdckcm@nus.edu.sg)

**Supplementary Material 1**. MEDLINE Search Strategy

| **#1** | exp Non-alcoholic Fatty Liver Disease/ or NAFLD.tw. or Hepatic Fat.tw. or ((liver or hepat*) adj3 (fatty or steatosis or steatoses)).tw. |
| --- | --- |
| **#2** | exp Fatty Liver/ or Non Alcoholic SteatoHepatitis.tw. or Non Alcoholic Steato hepatitis.tw. or ((fatty adj3 (liver* or hepat*)) or steatohepat* or NAFL* or NASH*).tw. |
| **#3** | exp Glucagon-Like Peptide 1/ or exp Exenatide/ or ((glucagon like peptide* or GLP 1 or GLP1) adj3 (analog* or agonist*)).tw. or (albenatide or albiglutide or beinaglutide or benaglutide or cotadutide or dulaglutide or efpeglenatide or exenatide or exendin-4 or exendin 4 or liraglutide or lixisenatide or pegapamodutide or taspoglutide or semaglutide or tirzepatide or LY307161).tw. |
| **#4** | (animals not humans).sh. |
| **#5** | #1 OR #2 |
| **#6** | #3 AND #5 |
| **#7** | #6 NOT #4 |

**Supplementary Material 2:** Changes in Hepatic Steatosis Markers

|  | **After GLP-1RA Treatment** | | **Comparisons against Controls** | | **Comparisons against TZD** | | **Comparisons against**  **DPP-4 Inhibitors** | | **Comparisons against Insulin-based Therapies** | | **Comparisons against Metformin** | |
| --- | --- | --- | --- | --- | --- | --- | --- | --- | --- | --- | --- | --- |
|  | **SMD (95% CI)** | **P-value** | **SMD (95% CI)** | **P-value** | **SMD (95% CI)** | **P-value** | **SMD (95% CI)** | **P-value** | **SMD (95% CI)** | **P-value** | **SMD (95% CI)** | **P-value** |
| Hepatic Fat Content | -1.05 (-1.62 to -0.48) | **<0.001** | -0.54 (-0.79 to -0.29) | **<0.001** | NA | NA | -0.04 (-0.59 to 0.51) | 0.88 | -0.66 (-0.97 to -0.36) | **<0.001** | -0.63 (-1.16 to -0.10) | **0.02** |

Legend:

Bolded outcome is statistically significant, p<0.05

GLP-1RA – Glucagon-Like Peptide-1 Receptor Agonist

TZD – Thiazolidinediones

NA – Not Applicable

**Supplementary Material 3:** Changes in Hepatic Fibrosis Markers

|  | **After GLP-1RA Treatment** | | **Comparisons against Controls** | | **Comparisons against TZD** | | **Comparisons against DPP-4 Inhibitors** | | **Comparisons against Insulin-based Therapies** | | **Comparisons against Metformin** | |
| --- | --- | --- | --- | --- | --- | --- | --- | --- | --- | --- | --- | --- |
|  | **SMD (95% CI)** | **P-value** | **SMD (95% CI)** | **P-value** | **SMD (95% CI)** | **P-value** | **SMD (95% CI)** | **P-value** | **SMD (95% CI)** | **P-value** | **SMD (95% CI)** | **P-value** |
| FIB-4 Index | -0.10 (-0.46 to 0.26) | 0.60 | 0.10 (-0.42 to 0.62) | 0.70 | NA | NA | 0.61 (0.05 to 1.17) | **0.03** | -0.16 (-0.52 to 0.20) | 0.39 | NA | NA |
| APRI Index | -0.68 (-1.24 to -0.18) | **0.02** | -0.21 (-1.04 to 0.62) | 0.62 | 0.23 (-0.36 to 0.81) | 0.45 | -0.62 (-1.14 to -0.10) | **0.02** | NA | NA | NA | NA |
| NAFLD Fibrosis Score | 0.12 (-0.45 to 0.69) | 0.68 | 0.12 (-0.28 to 0.51) | 0.56 | NA | NA | 0.23 (-0.32 to 0.78) | 0.41 | 0.00 (-0.57 to 0.57) | 1.00 | NA | NA |
| AST/ALT Ratio | 1.65 (1.33 to 1.97) | **<0.001** | 1.40 (1.11 to 1.68) | **<0.001** | NA | NA | NA | NA | NA | NA | 1.40 (1.11 to 1.68) | **<0.001** |

Legend:

Bolded outcome is statistically significant, p<0.05

GLP-1RA – Glucagon-Like Peptide-1 Receptor Agonist

TZD – Thiazolidinediones

NA – Not Applicable

**Supplementary Material 4:** Changes in Liver Function Test

|  | **After GLP-1RA Treatment** | | **Comparisons against Controls** | | **Comparisons against TZD** | | **Comparisons against DPP-4 Inhibitors** | | **Comparisons against Insulin-based Therapies** | | **Comparisons against Metformin** | |
| --- | --- | --- | --- | --- | --- | --- | --- | --- | --- | --- | --- | --- |
|  | **SMD (95% CI)** | **P-value** | **SMD (95% CI)** | **P-value** | **SMD (95% CI)** | **P-value** | **SMD (95% CI)** | **P-value** | **SMD (95% CI)** | **P-value** | **SMD (95% CI)** | **P-value** |
| Total Bilirubin | -5.83 (-7.01 to -4.66) | **<0.001** | -0.33 (-0.84 to 0.18) | 0.20 | NA | NA | NA | NA | -0.33 (-0.84 to 0.18) | 0.20 | NA | NA |
| AST | -1.46 (-2.22 to -0.79) | **<0.001** | -0.41 (-1.23 to 0.41) | 0.33 | 0.62 (0.03 to 1.22) | **0.04** | -0.04 (-0.98 to 0.90) | 0.94 | -0.57 (-1.14 to 0.00) | 0.05 | -0.08 (-0.48 to 0.33) | 0.71 |
| ALT | -1.69 (-2.32 to -1.07) | **<0.001** | -0.63 (-1.32 to 0.06) | 0.07 | 1.04 (0.42 to 1.67) | **<0.01** | 0.08 (-0.29 to 0.46) | 0.67 | -0.96 (-1.79 to -0.14) | **0.02** | -0.66 (-1.24 to -0.08) | **0.03** |
| GGT | -2.10 (-3.16 to -1.04) | **<0.001** | -0.81 (-1.87 to 0.26) | 0.14 | 0.69 (0.09 to 1.29) | **0.03** | 0.22 (-0.29 to 0.73) | 0.39 | -1.10 (-2.28 to 0.07) | 0.07 | -1.04 (-1.44 to -0.65) | **<0.001** |

Legend:

Bolded outcome is statistically significant, p<0.05

GLP-1RA – Glucagon-Like Peptide-1 Receptor Agonist

TZD – Thiazolidinediones

NA – Not Applicable

**Supplementary Material 5:** Changes in Body Composition

|  | **After GLP-1RA Treatment** | | **Comparisons against Controls** | | **Comparisons against TZD** | | **Comparisons against DPP-4 Inhibitors** | | **Comparisons against Insulin-based Therapies** | | **Comparisons against Metformin** | |
| --- | --- | --- | --- | --- | --- | --- | --- | --- | --- | --- | --- | --- |
|  | **SMD (95% CI)** | **P-value** | **SMD (95% CI)** | **P-value** | **SMD (95% CI)** | **P-value** | **SMD (95% CI)** | **P-value** | **SMD (95% CI)** | **P-value** | **SMD (95% CI)** | **P-value** |
| BMI | -0.98 (-1.45 to -0.51) | **<0.001** | -1.01 (-1.49 to -0.52) | **<0.001** | -0.92 (-1.54 to -0.31) | **<0.01** | -0.44 (-0.82 to -0.07) | **0.02** | -1.93 (-3.37 to -0.48) | **<0.01** | -0.58 (-0.81 to -0.34) | **<0.001** |
| Waist Circumference | -1.31 (-2.47 to -0.15) | **0.03** | -1.22 (-2.22 to -0.22) | **0.02** | NA | NA | -0.05 (-0.60 to 0.50) | 0.85 | -1.84 (-3.43 to -0.25) | **0.02** | -0.29 (-0.81 to 0.23) | 0.27 |
| Hip Circumference | -2.39 (-3.06 to -1.73) | **<0.001** | -3.71 (-4.55 to -2.87) | **<0.001** | NA | NA | NA | NA | -3.71 (-4.55 to -2.87) | **<0.001** | NA | NA |
| Waist-to-Hip Ratio (WHR) | -0.69 (-1.23 to -0.16) | **0.01** | -0.18 (-1.30 to 0.94) | 0.75 | NA | NA | NA | NA | -0.53 (-0.88 to -0.18) | **<0.01** | 0.17 (-2.12 to 2.47) | 0.88 |
| Subcutaneous Adipose Tissue | -0.32 (-0.68 to 0.05) | 0.09 | -0.76 (-1.21 to -0.32) | **<0.01** | NA | NA | -0.52 (-1.08 to 0.04) | 0.07 | -0.90 (-1.58 to -0.22) | **<0.01** | NA | NA |
| Visceral Adipose Tissue | -0.57 (-0.94 to -0.20) | **<0.01** | -0.66 (-1.03 to -0.28) | **<0.01** | NA | NA | -0.33 (-0.88 to 0.23) | 0.25 | -0.80 (-1.19 to -0.41) | **<0.001** | NA | NA |

Legend:

Bolded outcome is statistically significant, p<0.05

GLP-1RA – Glucagon-Like Peptide-1 Receptor Agonist

TZD – Thiazolidinediones

NA – Not Applicable

**Supplementary Material 6:** Changes in Metabolic Parameters

|  | **After GLP-1RA Treatment** | | **Comparisons against Controls** | | **Comparisons against TZD** | | **Comparisons against DPP-4 Inhibitors** | | **Comparisons against Insulin-based Therapies** | | **Comparisons against Metformin** | |
| --- | --- | --- | --- | --- | --- | --- | --- | --- | --- | --- | --- | --- |
|  | **SMD (95% CI)** | **P-value** | **SMD (95% CI)** | **P-value** | **SMD (95% CI)** | **P-value** | **SMD (95% CI)** | **P-value** | **SMD (95% CI)** | **P-value** | **SMD (95% CI)** | **P-value** |
| Fasting Glucose | -2.03 (-3.40 to -0.65) | **<0.01** | -0.20 (-0.39 to -0.01) | **0.04** | -0.04 (-0.63 to 0.54) | 0.88 | -0.51 (-1.00 to -0.02) | **0.04** | -0.22 (-0.49 to 0.05) | 0.11 | -0.21 (-0.70 to 0.27) | 0.39 |
| 2h Post-prandial Glucose | -2.15 (-3.31 to -0.98) | **<0.001** | -0.56 (-0.75 to -0.38) | **<0.001** | NA | NA | -0.15 (-0.70 to 0.40) | 0.60 | -0.74 (-1.16 to -0.33) | **<0.001** | -0.62 (-0.85 to -0.38) | **<0.001** |
| HbA1c | -2.17 (- 3.39 to -0.94) | **<0.01** | -0.02 (-0.18 to 0.14) | 0.80 | 0.16 (-0.42 to 0.75) | 0.59 | 0.29 (-0.26 to 0.83) | 0.30 | -0.37 (-0.62 to -0.11) | **<0.01** | 0.02 (-0.21 to 0.25) | 0.87 |
| HOMA-IR | -1.04 (-1.35 to -0.73) | **<0.001** | -0.19 (-0.45 to 0.07) | 0.16 | NA | NA | -0.56 (-1.12 to 0.00) | 0.05 | -0.46 (-1.03 to 0.18) | 0.12 | -0.02 (-0.28 to 0.23) | 0.86 |
| Total Cholesterol | -0.70 (-1.38 to -0.02) | **0.04** | -0.20 (-0.67 to 0.28) | 0.42 | NA | NA | 0.08 (-0.47 to 0.63) | 0.77 | -0.15 (-0.52 to 0.21) | 0.41 | -0.00 (-0.23 to 0.23) | 0.99 |
| Triglycerides | -0.84 (-1.44 to -0.24) | **<0.01** | -0.22 (-0.61 to 0.17) | 0.27 | 0.18 (-0.41 to 0.76) | 0.56 | -0.00 (-0.37 to 0.37) | 1.00 | -0.13 (-0.39 to 0.12) | 0.31 | -0.22 (-0.67 to 0.24) | 0.35 |
| FFA | -0.62 (-1.10 to -0.14) | **0.01** | 0.22 (-0.25 to 0.68) | 0.36 | NA | NA | NA | NA | 0.21 (-0.25 to 0.68) | 0.36 | NA | NA |
| HDL | -0.21 (-0.90 to 0.48) | 0.55 | -0.18 (-0.57 to 0.21) | 0.37 | NA | NA | 0.20 (-0.35 to 0.76) | 0.47 | -0.21 (-0.64 to 0.22) | 0.34 | 0.00 (-0.22 to 0.23) | 0.97 |
| LDL | -0.34 (-0.82 to 0.14) | 0.17 | -0.06 (-0.61 to 0.50) | 0.84 | 0.00 (-0.58 to 0.58) | 1.00 | 0.38 (-0.07 to 0.83) | 0.10 | -0.18 (-0.87 to 0.52) | 0.62 | 0.17 (-0.26 to 0.59) | 0.44 |
| Systolic Blood Pressure | -0.66 (-0.96 to -0.35) | **<0.001** | -0.25 (-0.46 to -0.04) | **0.02** | NA | NA | -0.20 (-0.75 to 0.35) | 0.48 | -0.37 (-0.62 to -0.11) | **<0.01** | 0.18 (-0.33 to 0.70) | 0.49 |
| Diastolic Blood Pressure | -0.33 (-0.59 to -0.07) | **0.01** | -0.07 (-0.32 to 0.17) | 0.56 | NA | NA | 0.52 (-0.04 to 1.07) | 0.07 | -0.18 (-0.44 to 0.08) | 0.17 | -0.16 (-0.68 to 0.35) | 0.54 |

Legend:

Bolded outcome is statistically significant, p<0.05

GLP-1RA – Glucagon-Like Peptide-1 Receptor Agonist

TZD – Thiazolidinediones

NA – Not applicable

**Supplementary Material 7:** Changes in Adipokines and Inflammatory Markers

|  | **After GLP-1RA Treatment** | | **Comparisons against Controls** | | **Comparisons against TZD** | | **Comparisons against DPP-4 Inhibitors** | | **Comparisons against Insulin-based Therapies** | | **Comparisons against Metformin** | |
| --- | --- | --- | --- | --- | --- | --- | --- | --- | --- | --- | --- | --- |
|  | **SMD (95% CI)** | **P-value** | **SMD (95% CI)** | **P-value** | **SMD (95% CI)** | **P-value** | **SMD (95% CI)** | **P-value** | **SMD (95% CI)** | **P-value** | **SMD (95% CI)** | **P-value** |
| Adiponectin | 0.84 (0.58 to 1.09) | **<0.001** | 0.75 (0.49 to 1.01) | **<0.001** | NA | NA | 0.61 (0.04 to 1.17) | **0.04** | 1.32 (0.69 to 1.95) | **<0.001** | 0.66 (0.40 to 0.93) | **<0.001** |
| CK-18 | -1.28 (-1.70 to -0.86) | **<0.001** | 0.80 (0.43 to 1.16) | **<0.001** | NA | NA | NA | NA | NA | NA | 0.80 (0.43 to 1.16) | **<0.001** |
| IL-6 | -0.74 (-1.33 to -0.16) | **0.01** | -0.38 (-1.10 to 0.35) | 0.31 | NA | NA | -0.01 (-0.56 to 0.54) | 0.96 | -0.75 (-1.34 to -0.17) | **0.01** | NA | NA |
| C-reactive Protein (CRP) | -2.02 (-2.36 to -1.68) | **<0.001** | -0.50 (-0.76 to -0.24) | **<0.001** | NA | NA | NA | NA | NA | NA | -0.50 (-0.76 to -0.24) | **<0.001** |

Legend:

Bolded outcome is statistically significant, p<0.05

GLP-1RA – Glucagon-Like Peptide-1 Receptor Agonist

TZD – Thiazolidinediones

NA – Not Applicable

**Supplementary Material 8:** Quality Assessment of Included Studies

| **Author, Year** | **Country** | **Study**  **Design** | **Selection** | **Comparability** | **Outcome** | **New-castle Ottawa Scale (NOS)**  **Total Score** |
| --- | --- | --- | --- | --- | --- | --- |
| Ohki et al, 2012 | Japan | Retrospective | 3 | 2 | 3 | 8 |
| Fan et al, 2013 | China | RCT | NA | NA | NA | NA |
| Shao et al, 2014 | China | RCT | NA | NA | NA | NA |
| Feng et al, 2017 | China | RCT | NA | NA | NA | NA |
| Tian et al, 2018 | China | Prospective | 3 | 2 | 3 | 8 |
| Zhang et al, 2018 | China | Retrospective | 3 | 2 | 3 | 8 |
| Yan et al, 2019 | China | RCT | NA | NA | NA | NA |
| Liu et al, 2020 | China | RCT | NA | NA | NA | NA |

Legend:

NA – Not Applicable

RCT – Randomised Controlled Trials
